# Improving Renal Protection in Chronic Kidney Disease Associated with Type 2 Diabetes: The Role of Finerenone

**DOI:** 10.2174/0118715303350851241021105850

**Published:** 2025-01-08

**Authors:** Pringgodigdo Nugroho

**Affiliations:** 1 Division of Nephrology and Hypertension, Department of Internal Medicine, Faculty of Medicine, Dr. Cipto Mangunkusumo Hospital, Universitas Indonesia, Jakarta, Indonesia

**Keywords:** Chronic kidney disease, type 2 diabetes mellitus, diabetic kidney disease, finerenone, nonsteroidal mineralocorticoid receptor antagonists, kidney replacement therapy

## Abstract

Chronic kidney disease (CKD) is a major complication of type 2 diabetes mellitus (T2D), which often leads to diabetic kidney disease (DKD). Traditional therapies, including renin-angiotensin-aldosterone system inhibitors and sodium-glucose cotransporter-2 inhibitors, are effective in slowing CKD progression. However, these approaches are insufficient to comprehensively inhibit mineralocorticoid receptor (MR) overactivation in the kidneys, which remains a significant driver of inflammation, fibrosis, and oxidative stress. These pathological processes accelerate kidney damage and cardiovascular complications. Finerenone-a nonsteroidal mineralocorticoid receptor antagonist-represents a new frontier in renal protection. Unlike steroidal mineralocorticoid antagonists (MRAs), finerenone offers a more selective MR blockade, reducing kidney inflammation and fibrosis without significantly raising serum potassium levels. Landmark trials have demonstrated the ability of finerenone to significantly reduce kidney and cardiovascular events in patients with T2D and CKD. Clinical evidence has highlighted finerenone as an effective option for slowing DKD progression while maintaining a favorable safety profile. Based on these findings, recent guidelines have incorporated finerenone as a recommended therapy for patients with T2D and CKD, emphasizing its role in reducing both renal and cardiovascular risks. This review provides a comprehensive overview of the available data to offer a deeper understanding of the potential of finerenone to transform CKD management for T2D patients.

## 1 INTRODUCTION

The study of chronic kidney disease (CKD) in diabetic individuals is crucial because of the widespread occurrence of diabetic kidney disease (DKD), which is the primary contributor to CKD among patients undergoing kidney replacement therapy (KRT) [1]. About 40% of individuals with diabetes are likely to experience DKD, and a significant portion of this population will advance to end-stage kidney disease (ESKD) [2]. DKD continues to be the primary cause of both CKD and ESKD globally [3]. It is associated with increased morbidity and mortality, primarily due to cardiovascular issues and advancing kidney disease necessitating KRT [4]. The occurrence of kidney disease among individuals with diabetes varies according to ethnicity, but generally increases in individuals with long-duration diabetes [5].

The risk of cardiovascular disease (CVD) increases significantly in individuals with CKD and diabetes mellitus (DM). Studies have shown that CKD correlates with a heightened risk of all-cause mortality and cardiovascular issues, observed within the general population and among individuals with DM [6, 7]. Furthermore, CKD is linked to a greater burden of cardiovascular risk factors, highlighting the importance of intensive cardiovascular risk reduction strategies for individuals with CKD [8, 9].

Similarly, DM is a known risk factor for CVD, including stroke, cardiac failure, and coronary heart disease [10, 11]. The risk of developing CVD increases for individuals with diabetic nephropathy-a microvascular complication of DM [6].

The current treatment approach for CVD, CKD, and DM has significantly improved patient outcomes. However, it remains challenging to fully overcome the complex interplay of these conditions. The existing treatment strategies primarily focus on managing blood pressure, glycemic control, and albuminuria to mitigate the progression of reno- and cardiovascular complications in diabetic individuals. Additionally, sodium-glucose transporter 2 (SGLT2) inhibitors are promising for reducing the risk of adverse cardiovascular and renal outcomes in patients with CKD, with or without concomitant cardiovascular disease [12-14]. Despite these advances, substantial proportions of the population remain at risk, and some uncertainty persists regarding the long-term efficacy of certain treatments, particularly in preventing end-stage kidney disease (ESKD) and reducing cardiovascular risk [13, 14].

Although the aim of current therapeutic strategies for managing CKD and type 2 DM (T2D) is to control risk factors and administer medications, such as SGLT2 inhibitors, the need for additional interventions remains critical. Thus, the emerging role of nonsteroidal mineralocorticoid receptor antagonists (nsMRAs) presents a novel avenue for addressing the complex interplay of cardiovascular and renal complications in this patient population [15-17]. In this article, the author delves into the potential benefits and efficacy of finerenone--an nsMRA--for managing T2D and CKD, exploring how this agent may complement existing treatments to improve patient outcomes and mitigate the progression of these intertwined conditions.

## 2 nsMRAs: A NEW FRONTIER

Mineralocorticoid receptor (MR) overactivation can induce inflammation and fibrosis in both kidney and cardiovascular tissues. Elevated MR activity is characteristic of both CKD and heart failure (HF) [18]. MR is an appealing therapeutic target due to its classical hemodynamic effects and its role in mediating inflammation and fibrosis in the renal and cardiac systems [19-21]. Under physiologic conditions, MR governs non stress glucocorticoid activation levels and regulates the modulation of ion and fluid transport, tropic and adaptive responses to injury, blood pressure control, and diverse biologic processes through the binding of aldosterone and cortisol. Upon binding, the MR allows transcription or repression of MR target genes [22]. There are certain conditions and pathology that cause MR overactivation, including, but not limited to, kidney and heart disease, oxidative stress, high salt load, obesity, and hyperglycemia [23]. This overactivation switches the natural physiological role of MR as a homeostatic regulator into a pathophysiological mediator through the stimulation of oxidative stress, inflammation, and fibrosis [24, 25].

The use of spironolactone as a steroidal mineralocorticoid receptor antagonist (MRA) has been a therapeutic strategy since the 1960s [22]. In the RALES trial, spironolactone exhibited a cardioprotective effect, reducing mortality risk by 30% compared to a placebo and improving ejection fractions [26]. The trial of spironolactone was prematurely terminated due to the efficacy of MRAs. However, the risk of hyperkalemia and sex hormone receptor-related side effects has limited its use. Hyperkalemia is a concern for patients with kidney dysfunction, as a low filtration rate can exacerbate the effect of potassium retention [18, 27].

In 2014, the results of the TOPCAT trial revealed that 15-45 mg/day of spironolactone did not lower the risk of primary composite events (cardiovascular (CV) death, aborted cardiac arrest, and/or hospital admission due to heart failure) in individuals with symptomatic heart failure with preserved ejection fraction (HFpEF) compared to a placebo. Moreover, it appeared to double the occurrence of hyperkalemia [28].

Currently, no large-scale outcome trials are being conducted to examine the long-term effects of steroidal MRAs on CKD progression in diabetic patients, primarily due to concerns about the development of hyperkalemia [29]. Steroidal MRAs for patients with T2D with or without CKD are recommended only as antihypertensive treatments for patients who are resistant to angiotensin-converting enzyme inhibitor (ACEi) / angiotensin II receptor blockers (ARBs) plus a diuretic [30, 31]. Steroidal MRAs are contraindicated for individuals with ESKD, and eplerenone is not recommended as an antihypertensive for patients with T2D and microalbuminuria due to the increased risk of hyperkalemia [22]. These constraints have driven the quest for alternative MR inhibition techniques that can selectively capitalize on cardiorenal benefits while reducing the risk of hyperkalemia and unwanted off-target adverse effects. To address these limitations, non-steroidal MRAs have been developed to provide targeted MR inhibition, potentially overcoming the challenges associated with traditional steroidal therapies.

## 3 MECHANISM OF ACTION OF nsMRAs

nsMRAs have promising cardiac and renoprotective properties for patients with CKD and HF [32]. nsMRAs work by targeting the physiological ligands of MR, which primarily include cortisol and aldosterone. MR is present in various cellular structures, mostly in the distal nephron and colon, but it is also expressed at lower levels in the heart, blood vessels, and central nervous system [33].

Aldosterone levels begin to rise when the glomerular filtration rate (GFR) is reduced by 50%. Higher serum aldosterone levels are associated with an increased risk of kidney disease progression [34], and aldosterone-mediated injury appears to be linked to a high salt intake, which disrupts the aldosterone-serum sodium balance. Administering aldosterone in mouse models causes the formation of reactive oxygen species (ROS) in various tissues, such as vascular smooth muscle cells, cardiomyocytes, and macrophages. ROS activate inflammatory transcription factors, such as AP-1 and NF-κB [35]. Aldosterone excess is associated with an increased risk of cardiovascular and renal complications, and elevated aldosterone levels can lead to hypertension, heart failure, and kidney damage by promoting sodium retention and fluid overload [36].

The aldosterone-MR complex advances the reabsorption of sodium and the excretion of potassium and hydrogen ions, highlighting the pivotal role of MR in controlling blood pressure, water and salt equilibrium, and circulating blood volume [37]. MR also plays a role in the inflammatory response by regulating cytokines and inflammatory mediator expression, activating inflammatory pathways, and facilitating inflammatory cell infiltration [38]. Excessive MR activation triggers ROS generation, inflammatory processes, and fibrogenesis, leading to myocardial hypertrophy, ventricular remodeling, renal damage, glomerular hypertrophy, glomerulosclerosis, and vascular damage [22, 39].

nsMRAs have cardiorenal protective effects through the targeted blockade of the MR-dependent promoter [40]. In contrast to steroidal MRAs, nsMRAs act through MR binding *via* an unstable receptor-ligand complex to reduce cofactor recruitment [41]. The delayed binding mechanism hinders the nuclear buildup of the MR-aldosterone complex and the recruitment of transcription cofactors downstream of the MR pathway, reducing the expression of proinflammatory and profibrotic factors [42].

**Fig. (1):**
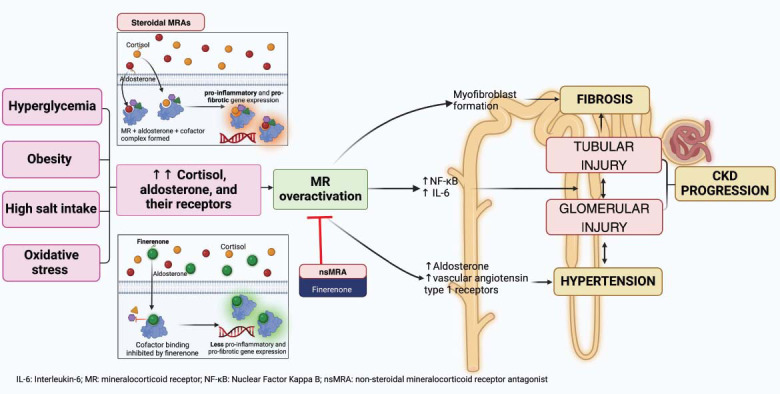
Pathological effects of mineralocorticoid receptor overactivation in the kidneys. (Created with BioRender.com).

In the heart, nsMRAs, including finerenone, have demonstrated a potent antifibrotic effect, resulting in improvements in cardiac hypertrophy and fibrosis [43]. In the kidneys, nsMRAs reduce endothelial cell (EC) apoptosis, inhibit smooth muscle cell proliferation, decrease leukocyte recruitment, and suppress the inflammatory response following vascular injury [44]. These actions promote endothelial repair and prevent adverse vascular remodeling. In general, MR activation increases the build-up of ROS in vascular smooth muscle cells (VSMCs) and ECs through the activation of NADPH oxidase. MR activation contributes to endothelial dysfunction, T-cell activation, macrophage infiltration, and cytokine buildup, all of which lead to fibrosis. MR activation also exacerbates podocyte effacement and injury, glomerular destruction, and endothelial impairment, all of which cause vascular remodeling [44]. nsMRAs significantly inhibit aldosterone binding to MR, inhibiting disease development and demonstrating renal-cardioprotective benefits (Fig. **1**) [44].

Based on the mechanism of action of nsMRAs, it is essential to explore the clinical evidence supporting the efficacy and safety of finerenone for relevant patient populations.

## 4 CLINICAL EVIDENCE REGARDING FINERE- NONE

Even with the use of renin-angiotensin system inhibitors and sodium-glucose cotransporter type 2 inhibitors, individuals with T2D and CKD continue to face elevated CV residual risk [45], which manifests in cardio-kidney-metabolic (CKM) syndrome [46]. MR overactivation can critically worsen CKM syndrome, primarily by causing inflammation and fibrosis, which traditional therapies do not address. The two largest pivotal phase 3 trials, FIDELIO-DKD and FIGARO-DKD, which highlighted the effectiveness of finerenone for patients with CKD and T2D, showed that finerenone reduces albuminuria and lowers the risk of CKD progression and CV events in such patients. The results of these trials were further analyzed using a prespecified pooled analysis called FIDELITY, the aim of which was to provide a complete assessment of the effects of finerenone on cardiorenal outcomes across a broad range of CKD severities in patients with T2D.

The FIDELIO-DKD trial was a randomized, double-blind, controlled trial conducted to assess the renovascular effects of finerenone in patients with CKD and T2D. The researchers enrolled 5,734 patients from 48 countries. To be eligible, patients had to have a UACR of 30-299 mg/g, an estimated glomerular filtration rate (eGFR) between 25 and < 60 mL/min/1.73 m^2^, an established diabetic retinopathy, or a UACR ≥ 300 mg/g with an eGFR of 25-74 mL/min/1.73 m^2^. All subjects received treatment with a maximum tolerated dose of RAS blockers. Patients were randomly assigned to groups that received either finerenone or a placebo in addition to standard treatment. The primary composite outcomes were renal failure, a ≥ 40% decline in eGFR compared to the baseline, or mortality from renal causes. The main secondary composite outcomes were death from cardiovascular causes, nonfatal myocardial infarction, nonfatal stroke, or hospital admission for heart failure [17].

During a median follow-up period of 2.6 years, primary composite events were observed in 17.8% of the participants in the finerenone group compared to 21.1% in the placebo group. Major secondary cumulative events occurred in 13% and 14.8% of the participants in the placebo and finerenone groups, respectively (HR 0.86, 95% CI 0.75-0.99, *p* = .03). Finerenone alleviated kidney failure, prolonged eGFR decreases by ≥ 40%, and reduced renal-related mortality. Regarding the secondary events, finerenone reduced the risk of cardiovascular death, nonfatal myocardial infarction, and hospitalization for heart failure compared to the placebo, but not nonfatal stroke. Finerenone resulted in a 31% lower UACR at 4 months, a decreased likelihood of kidney failure and renal-related mortality, and a ≥ 57% delayed eGFR decline. The combined rate of adverse events was comparable between the finerenone and placebo groups. However, individuals receiving finerenone had higher rates of hyperkalemia (18.3% *vs.* 9.0%) and therapy discontinuation due to hyperkalemia (2.3% *vs.* 0.9%). There were no reported incidents of fatal hyperkalemia. Finerenone reduced systolic blood pressure by around 2-3 mmHg compared to the placebo but had no effect on HbA1c or body weight [17].

The FIGARO-DKD was a multinational randomized controlled trial (RCT) conducted to investigate the impact of finerenone on cardiovascular outcomes in patients with established CKD and T2D. The trial included 7,437 individuals with T2D and CKD from 48 countries. Eligible patients had to have a UACR of 30-299 mg/g and an eGFR of 25-90 mL/min/1.73 m^2^ (stage 2-4 CKD) or a UACR of 300-5,000 mg/g and an eGFR ≥ 60 mL/min/1.73 m^2^ (stage 1-2 CKD). All patients were given a maximum dose of renin-angiotensin system (RAS) inhibition (that would not cause intolerable adverse effects). The patients were randomized 1:1 to receive either oral finerenone or a placebo. The primary events were cardiac mortality, nonfatal myocardial infarct, nonfatal cerebrovascular event (stroke), and hospital admission due to heart failure. The key secondary events were kidney failure, a ≥ 40% decline in eGFR from baseline, or death due to renal causes [17, 47].

Over a median follow-up of 3 years and 4 months, primary events were observed in 12.4% of the finerenone group *vs.* 14.2% of the placebo group due to finerenone lowering hospitalization rates for heart failure. The number needed to treat (NNT) with finerenone to prevent one primary outcome event was 47. Regarding secondary kidney composite events, the incidence was 9.5% with finerenone and 10.8% with the placebo, although neither reached statistical significance [47]. However, an exploratory analysis showed that finerenone reduced kidney composite events, including a sustained ≥ 57% decrease in eGFR. The cardiovascular benefits of finerenone were consistent, regardless of the baseline UACR and eGFR categories. The effects were also consistent, irrespective of background therapy with SGLT2 inhibitors or GLP-1 receptor agonists. The general incidence of adverse events was comparable for both groups. Finerenone was linked to higher treatment termination rates due to hyperkalemia (1.2%) than the placebo (0.4%). Finerenone led to a small increase in serum potassium compared to the placebo (a difference of 0.16 mmol/L) and modest reductions in blood pressure, with no difference in glycemic control or body weight [48]. This study extended the findings of the FIDELIO-DKD trial and established finerenone as a treatment option for reducing cardiovascular risk across a broad spectrum of CKD stages in patients with T2D. Along with SGLT2i and potentially GLP-1RAs, finerenone may substantially improve cardiorenal outcomes in this high-risk patient population.

The FIDELITY study was a prespecified pooled study of the FIDELIO-DKD and FIGARO-DKD trials. The focus of the analysis was specifically on the effects of finerenone on kidney outcomes. FIDELITY included 13,026 patients from FIDELIO-DKD and FIGARO-DKD with UACRs of 30-5,000 mg/g, eGFRs ≥ 25 mL/min/1.73 m^2^, and optimized treatment with an RAAS inhibitor. Patients were allocated 1:1 to a finerenone or placebo group and followed for a median of 3 years. The primary result was a combination of time to kidney failure, constant eGFR decline of ≥ 57% from baseline, or renal-cause mortality. Secondary events were ESKD and changes in UACR. Safety was evaluated using adverse event reporting [49].

Finerenone substantially lowered primary composite renal events by 23% compared to the placebo (HR 0.77, 95% CI 0.67-0.88). The 3-year absolute risk decrease was 1.7% (95% CI 0.7-2.6%), with a NNT of 60. Finerenone reduced the nonfatal outcomes, such as a 20% reduction in ESKD, and a consistent eGFR decrease of ≥ 57%. The effect of finerenone on primary renal events was consistent across the baseline eGFR and UACR categories. Patients with eGFRs < 45 mL/min/1.73 m^2^ and UACR ≥ 300 mg/g had the highest risk and received the most absolute benefit. In the UACR ≥ 300 mg/g subgroup, there was a NNT of 44. At 4 months, finerenone reduced the UACR by 32% compared to the placebo, with comparable reductions in UACR in the 30-300 and > 300 mg/g subgroups. The eGFR slope from month 4 to the end of therapy was substantially slower with finerenone than with the placebo (-2.5 *vs.* -3.7 mL/min/1.73 m^2^). Overall, finerenone and the placebo had equal adverse event rates. Finerenone was associated with higher rates of severe hyperkalemia, leading to treatment cessation, particularly in patients with eGFRs < 60 mL/min/1.73 m^2^. However, hyperkalemia resulting in hospitalization was infrequent (≤ 1.4%). The rates of acute kidney injury and deteriorating renal function were minimal and comparable across groups [49].

In this large, prespecified pooled analysis of patients with CKD and T2D, finerenone significantly reduced the risk of CKD progression and delayed the onset of ESKD compared to the placebo. The renal benefits were constant across the range of baseline CKD severities, with the greatest absolute benefits for those with more advanced diseases. Finerenone also significantly reduced albuminuria. The safety profile was acceptable, with low rates of treatment discontinuation due to hyperkalemia and no increased risk of acute kidney injury. These results support the use of finerenone, in addition to an optimized RAS blockade, to slow CKD progression in patients with T2D across a broad range of CKD stages, as shown in Table **1**.

The ARTS [50] trial reported that finerenone and spironolactone reduced natriuretic peptides and albuminuria to a similar extent. However, the risk of hyperkalemia was lower with finerenone than with spironolactone. The ARTS-HF [51] trial included 1,066 people with HF, reduced ejection fractions, and CKD and/or diabetes. This trial showed that finerenone and eplerenone similarly reduced natriuretic peptides, associated with a trend toward fewer major adverse cardiovascular events (MACE) (death from any cause, CV hospitalization, or emergency room visits for worsening HF up to day 90), which reached statistical significance on day 90 in the finerenone 10-20 mg group. ARTS-DN randomized 823 people with T2D and albuminuria (urinary albumin-creatinine ratios > 30 mg/g) who received treatment with ACE inhibitors or ARBs. Finerenone (at dosages of 7.5, 10, 15, and 20 mg orally daily) significantly reduced albuminuria compared to a placebo after 90 days of treatment [16].

Zheng *et al.* conducted a meta-analysis of four RCTs that included 13,945 patients. They reported that patients treated with finerenone showed significant decreases in UACR from baseline, indicating that finerenone could slow the progression of CKD by reducing albuminuria in diabetes patients. The finerenone group had a higher risk of hyperkalemia than the placebo group; however, there was no difference in the risk of overall adverse events [52].

## 5 FINERENONE *VERSUS* SPIRONOLACTONE

Steroidal MRAs, such as spironolactone, improve survival rates following heart failure and slow kidney disease progression, but they can cause side effects, such as hyperkalemia and gynecomastia, due to nonselective receptor binding. In contrast, nsMRAs, including finerenone, offer greater MR selectivity, reducing these side effects while providing similar cardiorenal protective benefits [25]. In this section, we discuss how steroidal and non-steroidal MRAs differ.

### 5.1 Tissue Distribution

Animal studies using [^14^C]-labeled eplerenone or [^3^H]-spironolactone showed a greater accumulation of drug-equivalent concentrations in kidney tissue compared to heart tissue in rodents [53]. However, finerenone demonstrated a balanced kidney-heart distribution in rats. Moreover, finerenone was not detected in brain tissues in preclinical studies conducted by Pitt *et al.,* suggesting that finerenone does not cross the blood-brain barrier [50].

### 5.2 Mode of Mineralocorticoid Receptor Antagonism

Molecular studies of the finerenone-MR complex have revealed that finerenone acts as a bulky, passive antagonist and is an effective MR blocker [54]. This distinctive binding mode influences its potency, selectivity, and interaction with nuclear cofactors. Additionally, its physicochemical properties, such as lipophilicity and polarity, play a key role in determining tissue penetration and distribution. Consequently, finerenone is more MR-selective and potent than its steroidal counterparts [25].

### 5.3 Pharmacokinetics

Among the steroidal MRAs, spironolactone has multiple active metabolites with long elimination half-lives [55], whereas finerenone (an nsMRA) has no active metabolites [56]. Renal excretion of unchanged finerenone is minimal [57], which is important for drugs that are intended for use in patients with chronic heart failure or DKD with reduced renal function. As a striking example, the AMBER trial, which evaluated spironolactone alongside patiromer or placebo in patients with resistant hypertension and advanced CKD (eGFR 25-45 mL/min/1.73 m^2^) reported that 20 of 23 patients (87%) had detectable spironolactone metabolites 1 week after the last spironolactone dose, 12 of 16 patients (75%) had detectable metabolites 2 weeks after the last spironolactone dose, and 4 of 11 patients (36%) had detectable metabolites 3 weeks after the last spironolactone dose [58].

### 5.4 Effects on Cardiac and Renal Damage Attenuation

In cardiorenal disease animal models, finerenone provided greater protection from heart and kidney damage than eplerenone at equinatriuretic dosages and decreased the renal gene expression of inflammatory/remodeling markers in a dose-dependent manner [53].

Differences in cofactor binding and ligand-mediated gene expression appeared to be the underlying molecular basis for the differences in cardiac and renal effects seen between steroidal and nsMRAs. Finerenone showed a different ligand-mediated cardiac gene expression profile from eplerenone in animal experiments, and this appeared to be mediated by selective MR cofactor modulation [59]. Finerenone was more effective than eplerenone in inhibiting the recruitment of transcriptional coactivators involved in the expression of genes associated with cardiac hypertrophy and fibrosis [60].

### 5.5 Hyperkalemia

Hyperkalemia (an elevated level of potassium in the blood) is a recognized potential side effect of MRAs [22]. Zhu *et al.* conducted a Bayesian network meta-analysis and reported that aldosterone antagonists pose the highest risk of hyperkalemia (98.8%) among antihypertensive drugs, followed by ARBs, renin inhibitors, diuretics, ACE inhibitors, and others, with calcium channel blockers (CCBs) posing the lowest risk (14.3%) [61].

Several studies have demonstrated that finerenone has a favorable hyperkalemia safety profile compared to traditional steroidal MRAs. For instance, the ARTS [50] and ARTS-HF [51] studies reported that finerenone led to smaller increases in serum potassium levels than spironolactone and eplerenone. The incidence of hyperkalemia was lower with finerenone than with spironolactone but comparable to eplerenone, suggesting a better risk-benefit ratio in terms of hyperkalemia risk for finerenone with similar efficacy.

A comparative post hoc analysis of 624 FIDELITY-TRH patients and 295 AMBER patients showed that finerenone had a 12% cumulative incidence of serum potassium ≥ 5.5 mmol/L after about 17 weeks, compared to 3% for a placebo, indicating some risk. However, finerenone still outperformed spironolactone in terms of safety, as the incidence of hyperkalemia in a comparative study was 35% for spironolactone + patiromer and 64% for spironolactone + a placebo at 12 weeks (*p* < .001). The FIDELITY-TRH study reported that finerenone reduced serum potassium to ≤ 5.5 mmol/L more quickly than the placebo. Meanwhile, in the AMBER study, spironolactone combined with patiromer took longer to achieve this reduction than spironolactone with the placebo [62]. Another meta-analysis reported that finerenone (10 mg/day) resulted in less hyperkalemia than spironolactone or eplerenone (25-50 mg/day) [63]. Similarly, the cardiovascular protection given by a dose of 10 mg/day of finerenone was superior to the protection provided by spironolactone or eplerenone at a dose of 25-50 mg/day [64]. These findings suggest that although finerenone carries a risk of hyperkalemia, it may be better tolerated than spironolactone, especially by patients with heart failure or CKD, making it a more suitable option for long-term management of cardiorenal diseases.

Despite these advantages, concerns remain regarding the potential of finerenone to increase hyperkalemia. Dimitrios *et al.* conducted a meta-analysis of cardiovascular outcome trials and reported that finerenone doubled the risk of hyperkalemia and significantly increased the risk of hospitalization due to hyperkalemia compared to a placebo. However, the same researchers noted that finerenone did not significantly impact the development of acute kidney injury (AKI) or hospitalizations due to AKI. They emphasized that although finerenone offers significant cardiovascular and renal benefits for patients with T2DM, careful hyperkalemia monitoring is essential [65].

In the following section, we will consider how hyperkalemia arises in finerenone treatment and how to prevent it.

## 6 MECHANISM OF HYPERKALEMIA IN FINE- RENONE TREATMENT

Under normal physiological conditions, approximately 90% of the potassium ions filtered by the glomerulus are reabsorbed in the proximal tubules and Henle loop. In the distal segment of the renal tubule and the collecting duct, the potassium ion concentration in the lumen is regulated by potassium, which remains non-reabsorbed in the proximal tubule, as well as by potassium secreted in the distal segment and the collecting duct. Potassium efflux in the cortical collecting duct occurs primarily through two channels: the renal outer medullary potassium (ROMK) channel and the Ca^(2+)^ -activated K^(+)^ (BK) channel [66]. The ROMK channel is the major excretory pathway under normal conditions, while the flow-dependent BK channel becomes more active under conditions of increased luminal flow [67]. Potassium excretion is further modulated by the renin-angiotensin-aldosterone system (RAAS), with aldosterone playing a key role in enhancing potassium excretion by binding to its receptor and promoting ROMK channel activity [68]. ROMK channels are sensitive to flow and hormonal regulation, as aldosterone upregulates ROMK expression and activity, ensuring efficient potassium secretion to maintain homeostasis, particularly when potassium levels rise, or sodium is reabsorbed [69].

RAAS inhibition often leads to hyperkalemia by disrupting aldosterone production or activity. Aldosterone secretion is influenced by three main factors: angiotensin II, elevated potassium levels, and adrenocorticotropic hormone (ACTH) [70, 71]. When aldosterone binds to MR in principal cells, it facilitates sodium reabsorption through the epithelial sodium channel (ENaC), which in turn enhances potassium excretion. Medications, such as ACE inhibitors, ARBs, and direct renin inhibitors, lower aldosterone secretion, reduce potassium excretion, and increase the risk of hyperkalemia. Additionally, MR antagonists and ENaC inhibitors act further downstream by either blocking the effect of aldosterone on MR or directly inhibiting ENaC, both of which increase aldosterone resistance [71].

Finerenone can induce hyperkalemia through its direct effects on the RAAS [72]. Under normal physiological conditions, aldosterone promotes sodium retention and potassium excretion in the distal nephrons of the kidneys by activating mineralocorticoid receptors [73]. Excess aldosterone secretion causes sodium and water retention, sympathetic nervous system activation, cardiac hypertrophy, fibrosis, and decreased baroreflex sensitivity [35]. Finerenone competitively inhibits these receptors, blocking aldosterone action, which leads to reduced sodium reabsorption and, consequently, diminished potassium excretion. This impaired potassium handling results in increased serum potassium levels, particularly in patients with CKD or heart failure, for whom renal function is already compromised. The risk of hyperkalemia is further heightened when finerenone is used in combination with other RAAS inhibitors, such as ACE inhibitors or ARBs, which also reduce potassium excretion [72].

## 7 ADDRESSING HYPERKALEMIA CONCERNS

Finerenone has a favorable profile for reducing the incidence of hyperkalemia compared to steroidal MRAs [74]. Despite the risk of hyperkalemia, the efficacy of finerenone in decreasing proteinuria and preserving renal function, coupled with its lower incidence of severe hyperkalemia compared to other MRAs, supports its use when carefully managed [15, 75].

Finerenone is not indicated for patients with eGFRs < 25 mL/min/m^2^ or serum potassium > 5.0 mEq/L. The starting dose of finerenone is 20 mg daily for an eGFR of ≥ 60 mL/minute/1.73 m^2^ and 10 mg daily for an eGFR of ≥ 25-59 mL/minute/1.73 m^2^. To maintain blood potassium levels of ≤ 4.8 mEq/L, the finerenone dose can be increased from 10 mg to 20 mg daily (maximum). If blood potassium surpasses 5.5 mEq/L, finerenone should be stopped and not restarted until blood potassium drops below 5.0 mEq/L [32].

Serum potassium and eGFR should be measured 4 weeks after starting finerenone and whenever the dose is increased. Regular monitoring allows the drug dosage to be adjusted based on serum potassium levels. Compared with no monitoring, regular monitoring of serum potassium can decrease the risk of hyperkalemia-related adverse events. Restricting the intake of potassium-rich foods is also effective in preventing hyperkalemia. Daily potassium intake should be limited to ≤ 2,000 mg/day for patients with Stage 3b CKD (eGFR between 30 and < 45 mL/min/1.73 m^2^) and to ≤ 1,500 mg/day for patients with Stage 4 or 5 CKD (eGFR < 30 mL/min/1.73 m^2^) [76].

Adjustment of the MR blocker dosage should be promoted alongside dietary potassium restriction, regular monitoring, the promotion of potassium excretion from the body using diuretics, the use of oral potassium adsorbent agents (such as calcium/sodium polystyrene sulfonate), the promotion of potassium redistribution from extracellular to intracellular spaces *via* intravenous administration of insulin and glucose, cell membrane stabilization *via* intravenous administration of calcium solution, and hemodialysis [77, 78].

## 8 FINERENONE GUIDELINES

Available evidence from robust clinical trials has led to the inclusion of finerenone in various clinical practice guidelines and consensus statements from leading medical organizations worldwide. The following sections provide an overview of the current guidelines for using nsMRAs to treat CKD patients with T2D.

### 8.1 Kidney Disease: Improving Global Outcomes (KDIGO) Guidelines

The KDIGO 2022 Clinical Practice Guidelines for Diabetes Management for T2D patients recommend a holistic approach to treating CKD. The guidelines emphasize the importance of a diverse treatment strategy that addresses numerous risk factors and pathophysiological pathways [79].

In the updated guidelines, KDIGO suggests using a nsMRA with reno- and cardiovascular benefits for patients with T2D with CKD, normal potassium concentrations, and albuminuria ≥ 30 mg/g regardless of treatment with the maximum tolerated dose of a RAS inhibitor [79]. This recommendation specifically applies to finerenone, as it is the only nsMRA that has demonstrated significant kidney and cardiovascular benefits for this patient population.

The guidelines assign a 2A grade to this recommendation, indicating that most patients prefer the recommended intervention and that there is high-quality evidence to support its use. This grading reflects the evidence supporting the use of finerenone for patients with CKD and T2D while acknowledging that individual patient preferences and clinical judgment should be considered when making treatment decisions.

### 8.2 American Diabetes Association (ADA) Guidelines

According to the 2024 ADA Standards of Care, patients with T2D with eGFRs of ≥ 25 mL/min/1.73 m^2^, normal serum potassium, and albuminuria ≥ 30 mg/g despite being treated with the maximum tolerable dose of a RAS inhibitor should be considered eligible to receive an nsMRA with proven renal or cardiovascular benefits [31, 80]. This recommendation specifically applies to finerenone and is graded as Level A. The guidelines for patients with eGFRs ≥ 20 mL/min/1.73 m^2^ propose using an SGLT2 inhibitor, a GLP-1 receptor agonist, or an nsMRA (if the eGFR is ≥ 25 mL/min/1.73 m^2^) to reduce cardiovascular risk in patients with T2D and CKD. Individuals with CKD and albuminuria are encouraged to receive nsMRA treatment if their eGFRs are ≥ 25 mL/min/1.73 m^2^. However, potassium levels should be checked when using nsMRAs. This recommendation is also graded as Level A, indicating clear evidence from well-conducted, generalizable RCTs that are adequately powered [80].

### 8.3 European Society of Hypertension (ESH) Guidelines

The European Society of Hypertension (ESH) guidelines, released in June 2023, recommend taking a comprehensive approach to the management of hypertension and its complications, including CKD, in patients with T2D. The ESH guidelines strongly recommend the use of finerenone for patients with T2D, CKD, and albuminuria if their eGFRs are ≤ 25 mL/min/1.73m^2^ and serum potassium levels are under 5.0 mmol/L. The recommendation is graded as Level IA, indicating the highest level of evidence and the strongest recommendation for use. Moreover, the guidelines recommend using a potassium binder to maintain potassium levels under 5.5 mEq/L in patients with CKD and hyperkalemia to allow for continued therapy with an RAS inhibitor or MRA. This recommendation is graded as Level IIB. The guidelines also discuss the concept of treatment sequencing, suggesting that finerenone can be used in combination with SGLT-2 inhibitors, either simultaneously or sequentially, based on a clinician’s judgment [81].

### 8.4 European Renal Best Practice (ERBP) Statement

The Board of the European Renal Association’s (ERA’s) ERBP statement summarizes current knowledge on MRAs for the treatment of patients with CKD and DM. The goal is to help professionals make daily decisions to manage patients with this condition. Finerenone is recommended for individuals with T2D, CKD, and albuminuria if their eGFRs are ≥ 25 mL/min/1.73m^2^ and serum potassium levels are < 4.8 mEq/L despite receiving a maximally tolerated dose of an RAS inhibitor. A potassium binder can be used to maintain near-normal serum potassium levels (< 5.5 mEq/L) for patients with hyperkalemia, thus facilitating the continuation of appropriate treatment with an RAS inhibitor or an MRA [82].

### 8.5 European Society of Cardiology (ESC) Guidelines

ESC guidelines, which are widely used by cardiologists, approach the management of CKD in T2D patients from a cardiovascular risk reduction perspective. These guidelines recommend statins to reduce cardiovascular risk and the use of nonsteroidal MRAs and SGLT-2 inhibitors to mitigate the risk of kidney failure in this patient population. The guidelines highlight the importance of employing interventions that can reduce adverse cardiovascular and adverse kidney events, such as treatment with finerenone, SGLT-2 inhibitors, and intensive blood pressure control [83].

However, the work of implementing these recommendations in everyday clinical practice and educating health-care providers across specialties remains an ongoing challenge. As additional evidence from ongoing trials on treatment with finerenone for nondiabetic CKD, type 1 diabetes, pediatric CKD, and heart failure patients becomes available, the treatment landscape for CKD is expected to evolve further. By staying abreast of the latest evidence and guidelines, clinicians can optimize CKD management for their patients, potentially slowing disease progression and improving both kidney and cardiovascular outcomes.

### 8.6 Other nsMRAs Undergoing Trials

Finerenone is currently the only approved nsMRA for patients with T2D and CKD who are at risk of cardiorenal disease. Other nsMRAs, such as esaxerenone and apararenone, are also being studied for similar indications, with both showing significant abilities to reduce albuminuria while maintaining favorable safety profiles. It is imperative to consider other nsMRAs that are currently being investigated in clinical trials.

Ocedurenone (KBP-5074) is another nsMRA under investigation for its potential blood pressure-lowering effects, which shows promise for managing hypertension in patients with CKD with comparable degrees of hyperkalemia compared to a placebo. Meanwhile, balcinrenone (AZD9977) is being explored for its protective effects in patients with CKD and HF, with the aim of providing cardiac function benefits. Balcinrenone seems to have MR modulatory effects rather than pure antagonistic effects. Both agents represent promising advances in targeting adverse cardiovascular and renal events, potentially expanding therapeutic options for patients at risk of heart and kidney complications. Detailed information is provided in Table **2** [84-87].

## 9 TREATMENT PARADIGM AND FUTURE DIRECTIONS

The current treatment paradigm for CKD in patients with T2D, as outlined in the various guidelines, can be visualized as a solid building founded on lifestyle changes and optimal glucose, blood pressure, and lipid control. This strong structure rests on four pillars: RAS inhibition, finerenone, SGLT-2 inhibitors, and GLP-1 RAs. Together, these four pillars deliver total reno- and cardiovascular protection for patients with CKD and T2D, each contributing its own mechanisms of action and therapeutic advantages. The addition of GLP-1 RAs as the fourth pillar further strengthens this treatment paradigm, as these agents have demonstrated significant cardiorenal protective effects in clinical trials [88-90]. The aim of this multidisciplinary approach, which targets several pathophysiological pathways and risk factors, is to halt CKD progression, reduce cardiovascular morbidity and mortality, and ultimately improve patient outcomes in this high-risk group. Although this represents substantial progress in the management of CKD for T2D patients, other patient categories still lack sufficient outcome data, such as those with nondiabetic CKD, type 1 diabetes, and heart failure.

Evidence suggests that combining SGLT2i and an nsMRA might have a synergistic effect due to their shared and distinct pathophysiological mechanisms, potentially reducing cardiorenal risk and albuminuria [91-94]. Additionally, the protective effect of SGLT2 inhibitors against hyperkalemia could allow patients to remain on finerenone treatment for longer [82]. However, further exploration is needed to fully understand the reno- and cardioprotective benefits of SGLT2 inhibitors for reducing adverse kidney and cardiovascular events, independent of glycemic status.

To fill these knowledge gaps, researchers are currently exploring the use of finerenone in diverse populations. The FIND-CKD study is being conducted to evaluate the efficacy and safety of finerenone for patients with nondiabetic CKD, with the primary outcome being a change in the chronic slope of eGFR [95]. The CONFIDENCE study is being conducted to assess the combination of finerenone with an SGLT-2 inhibitor compared to either therapy alone [96]. If CONFIDENCE demonstrates an additive effect with dual finerenone and SGLT2i therapy and acceptable safety, simultaneous therapy could be used to further slow kidney disease progression and provide long-term benefits for people with CKD and T2D.

The FINE-REAL trial is a noninterventional observational study designed to provide insights into the use of finerenone (10 mg and 20 mg) in a real-life clinical setting for patients with CKD and T2D [97]. The FINE-ONE researchers are investigating the use of finerenone for patients with type 1 diabetes [98], while FIONA is a phase III study to evaluate finerenone in conjunction with ACEi or ARB for pediatric patients with CKD and proteinuria [99]. The EFFEKTOR study is a pioneering multicenter randomized, double-blind, placebo-controlled clinical trial to assess the feasibility, tolerance, safety, and efficacy of finerenone for kidney transplant recipients [100].

In addition to CKD-focused trials, the MOONRAKER trial program is investigating the utility of finerenone for heart failure patients. The FINEARTS trial is assessing the use of finerenone for patients with heart failure with preserved ejection fractions (EF > 40%) [101]. Other ongoing trials in the MOONRAKER program include REDEFINE [102], CONFIRMATION [103], and FINALITY-HF [104], the collective aim of which is to obtain evidence for the use of finerenone for various heart failure populations that are likely to have concomitant CKD.

The development of other nsMRAs, such as esaxerenone, apararenone, ocedurenone, and balcinrenone, shows great potential for improving outcomes for patients with CKD at increased risk of cardiorenal diseases. These therapies offer significant benefits, particularly for reducing albuminuria, with comparable safety profiles. As the prevalence of cardiorenal diseases increases, continued research on nsMRAs is vital for optimizing treatment and improving patient outcomes, with a focus on slowing disease progression and minimizing cardiovascular risks.

## CONCLUSION

Finerenone represents a significant advance in the treatment of CKD in patients with T2D, offering both kidney and cardiovascular protection. Finerenone has consistently shown efficacy in decreasing renal and cardiovascular outcomes in patients with CKD and T2D in multiple trials. The FIDELIO-DKD, FIGARO-DKD, and FIDELITY researchers demonstrated the dual function of finerenone in kidney and cardiovascular protection, with the specific focus (renal *vs.* cardiovascular) varying by population and disease stage. The ARTS, ARTS-HF, and ARTS-DN studies have validated its advantages for heart failure and diabetic nephropathy patients while emphasizing its superior safety profile compared to steroidal mineralocorticoid receptor antagonists, particularly in terms of hyperkalemia.

The inclusion of finerenone in multiple guidelines underscores the strength of the supporting evidence. As healthcare providers work to implement these recommendations in clinical practice, finerenone has the potential to enhance outcomes for the increasing global population of patients with CKD and T2D. Ongoing research will continue to refine our understanding of the role of finerenone in the management of cardiorenal diseases and may lead to further updating of clinical practice guidelines.

This review is significant because it addresses a key gap in the management of CKD for individuals with T2D by investigating the impact of finerenone. We not only compared finerenone to classic steroidal MRAs, such as spironolactone, but also considered other nsMRAs that are currently undergoing trial, including esaxerenone, aparerenone, ocendurenone, and balcinrenone. Furthermore, we looked beyond CKD and T2D to highlight the broader cardiorenal benefits of nsMRAs.

Although we have made comparisons between finerenone and steroidal MRAs, head-to-head clinical trial data are still insufficient. Hence, a definitive conclusion about the superiority of finerenone cannot be drawn. Variability in patient populations could also have affected the generalizability of the findings.

Lastly, some future research directions include (but are not limited to 1) assessing the long-term efficacy and safety of finerenone beyond trial durations to provide insights into sustained benefits, potential risks, and or cardiovascular safety in broader populations, such as elderly, ethnic, and racial groups, or those vulnerable to CKD; 2) evaluating the impact of finerenone on nondiabetic CKD populations; 3) determining the mechanism of action of finerenone at different stages of CKD to understand its renoprotective effects; 4) investigating combination therapy with SGLT2i or GLP-1RA to enhance patient outcomes and minimize adverse effects; and 5) considering the cost-effectiveness and healthcare utilization of finerenone.

## Figures and Tables

**Table 1:** Finerenone hallmark trials.

Trial Design	Key Inclusion Criteria	Key Baseline Characteristics	Key Efficacy Outcomes	Key Safety Outcomes
FIDELIO-DKD [17] (N = 5,734) Phase 3 multinational RCT Median follow-up: 2 years and 6 months	• Patients with T2D aged ≥ 18 years • CKD (UACR 30-300 mg/g, eGFR 25-59 ml/min/1.73 m^2^, and retinopathy, **OR** UACR 300- 5,000 mg/g, and eGFR 25-74 ml/ min/1.73 m^2^) • Max. tolerated ACEi/ARB dose • Serum potassium ≤ 4.8 mEq/L	• Mean age: 65.6 years • Mean T2D duration: 16.6 years • Mean eGFR: 44.3 ml/min/1.73 m^2^ • Median UACR: 852 mg/g • Mean HbA1c: 7.7% • Mean SBP: 138 mmHg • Mean serum potassium: 4.4 mEq/L	Primary composite events: Renal failure, sustained eGFR decrease ≥ 40% from baseline, or renal-cause mortality: • Finerenone: 17.8% • Placebo: 21.1% Secondary composite events: Death from cardiovascular causes, myocardial infarction (MI), stroke, or hospital admission for HF: • Finerenone: 13.0% • Placebo: 14.8%	AE of hyperkalemia: • Finerenone: 446 (15.8%) • Placebo: 221 (7.8%) Serum potassium ≥ 5.5 mmol/L: • Finerenone: 21.7% • Placebo: 9.8% Serum potassium of ≥ 6.0 mmol/L: • Finerenone: 4.5% • Placebo: 1.4% Treatment discontinuation due to hyperkalemia: • Finerenone: 64 (2.3%) • Placebo: 25 (0.9%)
FIGARO-DKD [47] (N = 7,437) Phase 3 multinational RCT Median follow-up: 3 years and 4 months	• Patients with T2D aged ≥ 18 years • CKD, defined as: UACR 30-299 mg/g and eGFR 25-90 ml/min/ 1.73 m^2^, or UACR 300-5,000 mg/g and eGFR ≥ 60 ml/ min/1.73 m^2^ • Maximally tolerated ACEi/ARB • Serum potassium ≤ 4.8 mEq/L	• Mean age: 64.1 years • Median UACR: 308 mg/g • Mean eGFR: 67.8 ml/min/1.73 m^2^ • Mean duration of T2D: 14.5 years • Mean HbA1c: 7.7% • Mean SBP: 136 mmHg • Mean serum potassium: 4.3 mEq/L	Primary composite events: Death from cardiovascular causes, MI, stroke, or hospitalization for HF: • Finerenone: 12.4% • Placebo: 14.2% Key secondary composite kidney events, a sustained decrease of ≥ 40% in eGFR from baseline, or renal cause mortality: • Finerenone: 9.5% • Placebo: 10.8%	Hyperkalemia: • Finerenone: 396 (10.8%) • Placebo: 193 (5.3%) Treatment discontinuation due to hyperkalemia: • Finerenone: 46 (1.2%) • Placebo: 13 (0.4%)

**Table 2:** Summary of nsMRA clinical trials.

**Trial Design**	**Key Inclusion Criteria**	**Key Baseline Characteristics**	**Key Efficacy Outcomes**	**Key Safety Outcomes**
**Esaxerenone**				
ESAX-DN [84](N = 455) Phase 3, 52-week, multicenter, randomized, double-blind, placebo-controlled study conducted in Japan to compare esaxerenone with a placebo	• Patients with T2D and hypertension aged ≥ 20 years • Microalbuminuria, UACR between 45 and < 300 mg/g • eGFR ≥ 30 ml/ min/1.73 m^2^ • Prior ACEi/ARB treatment	• Mean age: 66 years • Mean eGFR: 69 ml/min/1.73 m^2^ • Mean UACR: 110 mg/g • Mean duration of T2D: 14 years • Mean HbA1c: 7.0% • Mean SBP: 140 mmHg • Mean serum potassium: 4.3 mmol/L	Primary event: • UACR remission: Esaxerenone (22%) *vs.* placebo (4%). Secondary events: • Change in UACR from baseline to end of treatment ≥ 30% • Esaxerenone (-58%) *vs.* placebo (8%). • Geometric least-squares mean ratio: Esaxarenone 1.08; placebo 0.42	Total number of treatment-emergent adverse events: • Esaxerenone: 528 • Placebo: 530 Increased AE of blood potassium: • Esaxerenone: 20 (9%) • Placebo: 5 (2%) Treatment discontinuation due to hyperkalemia: • Esaxerenone: 10 (4%) • Placebo: 1 (0.4%) Conclusion: The addition of esaxerenone to existing RASi is beneficial for reducing the progression of albuminuria.
**Apararenone**				
MT-3995 [85] (N = 293) Two-part, Phase 2 study conducted in Japan Part A: 24-week, randomized, double-blind, placebo-controlled, dose-response study (2.5-10 mg/day apararenone) Part B: 28-week, randomized, open-label, uncontrolled extension study	• Patients with T2D aged 20-75 years • CKD, defined as UACR ≥ 50 mg/g in casual urine sample, median UACR between 50 and < 300 mg/g, and eGFR ≥ 30 ml/min/1.73 m^2^ • HbA1c ≤ 10.5% • SBP < 160 mmHg • DBP < 100 mmHg	• Mean age: 60-63 years • Mean eGFR: 71-78 ml/min/1.73 m^2^ • Median UACR: 109-132 mg/g • Mean duration of T2D: 13-15 years • Mean HbA1c: 7.0-7.3% • Mean SBP: 133-137 mmHg • Use of ACEi/ARB: 64% • Mean serum potassium: 4.2-4.3 mmol/L	Primary event: UACR as a percentage of baseline level at week 24: • 2.5 mg: 62.9% (95% CI 54.6-72.5 • 5 mg: 50.8% (95% CI 44.1-58.4) • 10 mg: 46.5% (95% CI 40.4-53.5) • Placebo: 113.7% (95% CI 98.5-131.2) *p* < .001 for all apararenone groups *vs.* a placebo	Part A (dose-response study) Five patients discontinued treatment due to hyperkalemia: • 2.5 mg: 2 (2.7%) • 5 mg: 0 • 10 mg: 3 (4.1%) • Placebo: 0 Part B (extension study) The AE of blood potassium increased: • 2.5 mg: 0 • 5 mg: 3 (4.7%) • 10 mg 1 (1.6%) Conclusion: A confirmed UACR lowering effect in T2D patients over 24 weeks; 52-week administration was safe and tolerable.
**Ocedurenone (KBP-5074)**				
BLOCK-CKD (N = 162) [86] Phase 2b, multicenter randomized, double-blind, placebo-controlled study KBP-5074 addition to standard treatment 1:1:1 placebo, KBP-5074 0.25 mg, KBP-5074 0.5 mg	• Adults aged 18-85 years of age • Stage 3B/4 CKD (GFR ≥ 15 and ≤ 44 ml/min) • Uncontrolled HTN (SBP ≥ 140 mmHg) • Maximally tolerated dose of ≥ 2 anti-HTN medications	• Mean age: 65.4 years • Mean eGFR: 31.9 ml/min/1.73 m^2^ • Mean SBP: 155.3 mmHg • UACR: • > 300: 85 (52.5%) • 30-300: 40 (24.7%) • < 30: 35 (21.5%) • Mean potassium: 4.38 mmol/L	Primary event: SBP change from baseline on day 84: • KBP-5074 0.25 mg: -7.0 (3.37) mmHg • KBP-5074 0.50 mg: -10.2 (3.32) mmHg	Hyperkalemia ≥ 5.6 < 6 mmol/L: • Placebo: 5 (8.8%) • KBP-5074 0.25 mg: 6 (11.8%) • KBP-5074 0.50 mg: 9 (16.7%) No hyperkalemia ≥ 6.0 mmol/L reported Conclusion: KBP-5074 lowers blood pressure (BP) with some risk of hyperkalemia.
**Balcinrenone (AZD9977)**				
NCT03682497 [87] Phase 1b, open-label, randomized, multicenter study. Assessing AZD9977 and spironolactone on K^+^ in HF and CKD.	• Adults ≥ 18 years • BMI < 40 kg/m^2^ • Clinical diagnosis of HF, LVEF ≥ 40% • eGFR 4070 ml/min/1.73 m^2^ • NTproBNP ≥ 125 pg/ml • Baseline serum potassium: 3.5-4.8 mmol/L • On a stable dose of loop diuretic plus either: ARB/ACEi	• Mean age: 73.0 years • Mean potassium: 4.49 mmol/L • Mean eGFR: 58 (14) ml/min/1.73 m^2^ • Mean UACR: N/A	Primary event: Relative change in serum potassium from baseline on day 28: -0.3% (-5.3-4.4%), *p* = .888. Geometric mean change: • AZD9977: 5.73%, increase 0.26 mmol/L • Spironolactone: 4.21%, increase 0.19 mmol/L	Adverse events: • AZD9977 (39.4%) • Spironolactone (17.1%) AE leading to discontinuation of treatment: • AZD9977 (3%) • Spironolactone (5.7%) No patients discontinued treatment due to hyperkalemia Conclusion: AZD9977 had favorable safety effects against spironolactone. It seemed to have MR modulatory effects rather than pure antagonistic effects.

## LIST OF ABBREVIATIONS

AKI: Acute Kidney Injury
CVD: Cardiovascular Disease
CKD: Chronic Kidney Disease
DKD: Diabetic Kidney Disease
EC: Endothelial Cell
GFR: Glomerular Filtration Rate
KRT: Kidney Replacement Therapy
MR: Mineralocorticoid Receptor
SGLT2: Sodium-Glucose Transporter 2
VSMCs: Vascular Smooth Muscle Cells
